# Retraction statement: microRNA‐497 inhibits cell proliferation and induces apoptosis by targeting YAP1 in human hepatocellular carcinoma

**DOI:** 10.1002/2211-5463.13510

**Published:** 2022-11-15

https://doi.org/10.1002/2211-5463.12032

The above article published online on 19 January 2016 in Wiley Online Library (wileyonlinelibrary.com), has been retracted by agreement between the Editor‐in‐Chief, John Wiley and Sons Ltd and the authors. The retraction has been agreed following an investigation based on allegations raised by a third party, which revealed that some images in this article are inappropriate duplications of images from previously published articles [1, 2, 3, 4]. Thus, the editors consider the conclusions of this manuscript substantially compromised.
